# Photocurable Polymeric Blends for Surgical Application

**DOI:** 10.3390/ma13245681

**Published:** 2020-12-12

**Authors:** Teresa Cernadas, Marta Santos, Sónia P. Miguel, Ilídio J. Correia, Patrícia Alves, Paula Ferreira

**Affiliations:** 1 CIEPQPF, Department of Chemical Engineering, University of Coimbra, P-3030 790 Coimbra, Portugal; mariac@eq.uc.pt (T.C.); marta.santos@uc.pt (M.S.); icorreia@fcsaude.ubi.pt (I.J.C.); palves@eq.uc.pt (P.A.); 2 CICS-UBI, Health Sciences Research Center, University of Beira Interior, P-6200 506 Covilhã, Portugal; spmiguel@ubi.pt; 3 CPIRN-IPG, Center of Potential and Innovation of Natural Resources, Polytechnic Institute of Guarda, Av. Dr. Francisco de Sá Carneiro, 6300-559 Guarda, Portugal

**Keywords:** photocuring, polymeric blends, bioadhesive, biocompatibility, antibacterial activity

## Abstract

The preparation of photocrosslinkable bioadhesives synthesized from oligomers of lactic acid and polycaprolactone (PCL), both functionalized with 2-isocyanoethyl acrylate (AOI), were studied. The obtained modified macromers of LA-AOI (mLA) and PCL-AOI (mCL) were chemically characterized by ^1^H NMR and used to formulate polymeric blends with different mass proportions, 1:1, 1:2 and 2:1, respectively. Subsequently, the produced blends were crosslinked, considering two UV irradiation times: 30 and 120 s. After their production, the thermal and mechanical properties of bioadhesives were assessed, where upon the rheology, gel content, hydrolytic degradation and dynamic contact angles were determined. Furthermore, the cytotoxic profile of bioadhesives was evaluated in contact with human dermal fibroblasts cells, whereas their antibacterial effect was studied monitoring *Escherichia coli* and *S. aureus* growth. Overall, flexible and resistant films were obtained, presenting promising features to be used as surgical bioadhesives.

## 1. Introduction

At the end of the 20th century, a new trend emerged regarding the development of polymeric materials with numerous scientific studies reporting the use of polymeric blends as potential new materials for biomedical applications [1], and more specifically, on their use in the preparation of bioadhesives [2].

In fact, polymer blending is recognized as an inexpensive procedure that enables the adjustment of the materials’ final properties, with a full set of tailored characteristics and improved specific features [1]. Generally, polymeric blends can be obtained by using one of two basic methods: blending melted or softened components, and/or by mixing solutions of components in the same solvent [3,4].

Among the synthetic aliphatic polyesters, two of the most frequently used polymers in biomedical applications are poly(lactic acid) (PLA) and polycaprolactone diol (PCL), both approved by the Food and Drug Administration [5,6,7]. PLA is a highly versatile biodegradable and biocompatible polymer [8] with a glass transition temperature (T_g_) above room temperature, making it a hard and brittle material [9,10]. PCL is a biocompatible semi-crystalline linear polyester known for its high permeability, slow degradation rate and low T_g_, rendering it resistant and tough [9]. In both cases, their structures present several aliphatic ester linkages susceptible to hydrolysis, not depending on the action of enzymes to undergo biodegradation and, therefore, precluding inflammatory responses [7]. Thus, the products of hydrolytic degradation can either be metabolized, through the inclusion in a cell’s metabolic pathway, or eliminated by renal secretion [11]. In both cases, physical–mechanical properties and biodegradation behavior can be tailored by blending, copolymerization or by changing the macromolecular architecture [12].

The work developed herein comprises polymeric blends based on low molecular weight (oligomers) of caprolactone (CL) and lactic acid (LA) that were further characterized and tested as possible materials for biomedical application. Both CL and LA oligomers were functionalized with 2-isocyanatoethyl acrylate (AOI), a vinylic isocyanate monomer. The choice of the AOI was based on the inexistence of reported research of this compound in the preparation of bioadhesive materials.

Three different stoichiometric proportions of functionalized PCL/LA oligomers mixtures were prepared and once the photocrosslinker Irgacure^®^2959 (Ir2959) was added to the solutions [13], well-defined crosslinked networks were obtained after two distinct irradiation times (30 and 120 s). Therefore, the chemical functionalization with AOI as the vinylic monomer allowed to obtain crosslinked films with curing times appropriate to surgical demands. The choice of Ir2959 as the photoinitiating agent was based on literature that reported this compound as biocompatible in several cell lines and under a broad range of tested concentrations [13].

The chemical and physical properties of photocrosslinked films were further characterized in order to demonstrate their suitability as UV-curable bioadhesives. Additionally, their biocompatibility as well as their antibacterial activity were assessed.

## 2. Materials and Methods

### 2.1. Materials

Polycaprolactone diol (PCL, Mn ≈ 530 g.mol^−1^) and Lactic Acid L(+) (LA, 80%) were acquired from Sigma-Aldrich (Sintra, Portugal). Diethyl ether (99%), 1,4-butanediol (99%) and anhydrous calcium chloride (96%) were purchased from ACROS organics. 2-hydroxy-1-[4-(2-hydroxyethoxy) phenyl]-2-methyl-1-propanone (Irgacure^®^ 2959, 97–99%) and 2-isocyanatoethyl acrylate (AOI, >98%)) were purchased from Ciba Specialty Chemicals (Groot-Bijgaarden, Belgium) and TCI (Zwijndrecht, Belgium), respectively. The deuterated chloroform used as solvent was obtained from Merck (Kenilworth, New Jersey, USA). No further purification was performed to any of these reagents. Fetal bovine serum (FBS) (free from any antibiotic) was obtained from Biochrom AG (Berlin, Germany). Normal Human Dermal Fibroblasts (NHDF) cells were purchased from PromoCell (Labclinics, S.A., Barcelona, Spain). 3-(4,5-Dimethylthiazol-2-yl)-2,5-diphenyltetrazolium bromide (MTT) was acquired from Alfa Aesar (Ward Hill, MA, USA). Amphotericin B, Dulbecco’s modified Eagle’s medium (DMEMF12) and Trypsin were obtained from Sigma-Aldrich (Sintra, Portugal). *Staphylococcus aureus* clinical isolate (*S. aureus,* ATCC 25923) and *Escherichia Coli* DH5 α (*E. coli,* DH5a) were purchased from ATCC (Teddington, UK) and used as model organisms to evaluate the antimicrobial properties of the adhesives.

### 2.2. Blending and Crosslinkg

#### 2.2.1. Lactic Acid Oligomers Synthesis

Oligomers based on lactic acid (oligLA) were synthesized through polycondensation reactions, under atmospheric pressure. The reaction of lactic acid with 1,4-butanediol, was performed in the stoichiometry of 6:1, respectively, in a round-bottom glass flask equipped with a water glass collector and placed in an oil bath at the temperature of 150 °C. Constant nitrogen flow and magnetic stirring (Labox, LBX H03D, 3 L, Labbox Labware S.L., Spain) were maintained throughout the reaction time of 9 h.

#### 2.2.2. Modification of Prepolymers with AOI

The modification of the oligLA and PCL was achieved by using AOI, a vinylic monomer, in two distinct reactions. The volume of AOI was determined to ensure total reaction of isocyanate groups. Diethyl ether (10 mL per 0.02 mol of oligomer) was used as solvent to dissolve AOI (0.04 mol) in the previous mixtures. Both reactions proceeded in a round-bottom flask at 60 °C, for 24 h, under reflux and magnetic stirring. The obtained functionalized macromers are further nominated as mLA (macromer of LA-AOI) and mCL (macromer of PCL-AOI).

#### 2.2.3. Preparation of Blends and Photocrosslinking

The blends were obtained through homogeneously mixing mLA and mCL, varying the mass proportions of each functionalized macromers. The obtained blends were designated as:

- LA1CL1: mLA and mCL in 1:1 ratio;
- LA2CL1: mLA and mCL in a 2:1 ratio;
- LA1CL2: mLA and mCL in a 1:2 ratio.

In order to prepare the blends, 4% (considering the carbon double bonds moles) of Irgacure^®^ 2959 (Ir2959) was added to the mixture and stirred under heat to obtain a homogeneous solution. Thereafter, these solutions were uniformly spread with a stainless-steel cylinder of 1 mm of thickness, and UV irradiated for 30 s and 2 min using a Multiband UV UVGL-48 with a wavelength of 356 nm from Mineral Light^®^ Lamp (UVP LLC, Upland, CA, USA). Transparent, uniform and flexible films were obtained and further characterized.

### 2.3. Characterization Techniques

#### 2.3.1. Nuclear Magnetic Resonance (NMR) Spectroscopy

mLA, mCL and the blends (LA1CL1, LA1CL2 and LA2CL1) chemical structure were confirmed by ^1^H NMR analysis. Deuterated chloroform (CDCl_3_) was selected as solvent and tetramethylsilane used as the internal reference. The spectra were obtained at room temperature in a Bruker Avance III 400 MHz, 9.4 Tesla Spectrometer (Brucker, Ettlingen, German).

#### 2.3.2. Rheological Studies

Blends viscosity was determined using a controlled stress rheometer (Haake, model RS1, Karlsruhe, Germany) with a plate/plate system PP20Ti geometry (titanium for the rotating part and stainless steel for the stationary part) connected to a temperature control recirculation bath (Haake Phoenix II, Karlsruhe, Germany). The rheological behavior of the materials was measured at 25 °C with a 0.2 mm GAP and the viscosities in function of the shear rate over time were attained. The power law parameters, fitted using the Ostwald-de Waele model, were obtained using the Haake RheoWin 4.20.005 software (Haake, vision, Karlsruhe, Germany).

#### 2.3.3. Gel Content

Solvent extraction was used to determine the gel content of the obtained films to qualitatively infer about the crosslinking degree. Briefly, dried films (20 × 10 × 1 mm) were weighed (*W_i_*), then immersed in diethyl ether (99%) sealed containers, under stirring and at room temperature, overnight. Films were then removed, dried and reweighed (*W_f_*). Three samples of each film were measured and results presented as mean and standard deviation. Gel content was determined according to Equation (1).

(1) $$\mathrm{Gel}\;\mathrm{content}\;\left(\%\right)=\;\left(\frac{W_{f}}{W_{i}}\right)\;\times 100$$

#### 2.3.4. Hydrolytic Degradation

Hydrolytic degradation of three dried samples of each prepared film, with dimensions of 20 × 10 × 1 mm, were weighed (*W_d,o_*). Then, they were immersed in Phosphate Buffer Saline solution (PBS, 0.01 M, pH 7.4) and incubated at 37 °C for six weeks. Films were removed over time (24 and 72 h and 1, 2, 3, 4, 5 and 6 weeks) from the PBS solution, washed with distilled water and dried under vacuum, at 37 °C, until constant weight (*W_d,t_*). The degradation degree was assessed from the determination of weight loss by using Equation (2).

(2) $$\mathrm{Weight}\;\mathrm{loss}\;\left(\%\right)=\;\frac{W_{d,0}{-W}_{d,t}}{W_{d,0}}\;\times 100$$

#### 2.3.5. Dynamic Contact Angles Measurements

A Dataphysics OCA-20 contact angle goniometer (DataPhysics Instruments, Filderstadt, Germany) was used to determine the dynamic contact angles of the films, using the sessile drop method. Briefly, 10 µL droplet of deionized water was mechanically dispensed on the surface of the films and its progress across time was recorded with a CCD video camera (DataPhysics Instruments, Filderstadt, Germany). Water contact angles were calculated using the equipment software. The results obtained are the mean values of three independent measurements.

#### 2.3.6. Thermal Analysis

Thermo gravimetric analysis (TGA) was performed for all materials (before and after crosslinking) in order to evaluate their thermal stability. A TGA equipment SDT Q500 from Thermal Analysis (TA) Instruments (New Castle, DE, USA) was used. Amounts of 5 to 10 mg of each material were heated to 600 °C with a heating rate of 10 °C/min, under nitrogen purge with a flow rate of 100 mL/min. Data processing and determination of the degradation temperatures were performed with the universal analysis 2000 software from TA Instruments.

#### 2.3.7. Cell Growth and Proliferation

Cell adhesion and proliferation of the Normal Human Dermal Fibroblasts (NHDF) in contact with crosslinked polymeric films were assessed. To accomplish such purpose, the films were positioned into 96-well plates and sterilized for 1h by UV irradiation. After that, cells were seeded at a density of 10 × 10^3^ cells per well. Along different timepoints, cell growth in the presence of the films was monitored using an Olympus CKX41 inverted light microscope equipped with an Olympus SP-500 UZ digital camera (Olympus Life Science, Waltham, MA, USA). To accomplish that, each well containing cells seeded in contact with films were photographed (at magnification of 100×) aiming to characterize the cell morphology of human fibroblasts.

Furthermore, the cytotoxic profile of the films was evaluated by MTT assay, following the guidelines established in ISO 10993-5. In brief, at different timepoints, the culture medium of each well was replaced by 50 µL of MTT (5 mg/mL PBS) (*n* = 5). After that, the plate was incubated for 4 h, at 37 °C, in a 5% CO_2_ atmosphere to promote the metabolic conversion of MTT. During this time of incubation, the yellow tetrazole salt is converted to purple formazan crystals by living cells and such process is proportional to the number of viable cells present in each well. Then, the insoluble formazan crystals produced were dissolved with 200 µL of DMSO (0.04 N) for 30 min. The absorbance at 570 nm was read in a microplate reader (Biorad × Mark microplate spectrophotometer). Cells cultured without materials were used as negative control (K^−^), while cells cultured with EtOH (96%) were used as positive control (K^+^).

#### 2.3.8. Antimicrobial Activity

The films antimicrobial properties were determined by using *S. aureus* and *E.coli* as Gram-positive and Gram-negative bacteria models, respectively. Initially, bacteria culture at a concentration of 1 × 10^8^ CFU/mL were dispensed onto agar plates [14,15]. Then, circular films (*n* = 3) were placed on the agar plate and incubated for 24 h, at 37 °C. Afterwards, the inhibitory halo diameter induced by the films were photographed and measured using ImageJ software (Scion Corp., Frederick, MD, USA). In addition, the characterization of the bacterial colonization at films’ surface was recorded by SEM.

To accomplish that, samples were initially fixed with 2.5% (*v/v*) glutaraldehyde for 4 h. Then, they were washed three times with PBS and subsequently dehydrated with increasing concentrations of EtOH (70%, 80%, 90% and 100%) and freeze-dried for 3 h. Finally, the samples were fixed onto aluminum stubs with Araldite glue and sputter-coated with gold using a Quorum Q150R ES sputter coater (Quorum Technologies Ltd., Laughton, East Sussex, UK). SEM images were finally acquired with different magnifications, using an acceleration voltage of 20 kV, in a Hitachi S-3400N Scanning Electron Microscope (Hitachi, Tokyo, Japan) [15,16].

## 3. Results and Discussion

### 3.1. Synthesis

The experimental synthesis procedures here reported allowed three different photocrosslinked materials to be obtained by blending the two macromers, mCL and mLA, in different mass proportions (1:1, 2:1 and 1:2).

Initially, both PCL and oligLA were modified with a functional agent, AOI, to introduce photocrosslinkable sites in the polymers chain. This reaction occurred between the hydroxylic (-OH) groups of the diol with the isocyanate groups of AOI (-NCO) resulting in urethane linkages (-NHCOO-). The experimental protocol was designed in accordance with the functional agent and the authors’ prior research work [2,17,18]. All blends obtained presented an apparent viscosity which led to infer a good behavior for in situ application.

**Figure 1:**
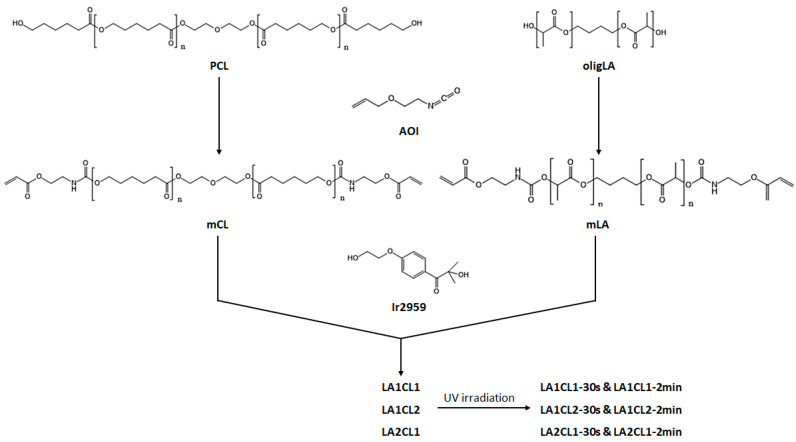
Schematic representation of the macromers synthesis and preparation of the three different blends.

Crosslinking were performed using a percentage of 4% of the carbon double bonds moles with a well-known biocompatible photoinitiator (Ir2959). Different irradiation times were tested and after 30 s crosslinked networks were obtained. However, a second irradiation time of 2 min was also tested in order to determine potential differences in the materials properties. It was found that the films with the highest amount of mLA were more flexible, while those that contained the highest amount of mCL became more resistant. A typical reaction scheme is presented in Figure 1.

### 3.2. ^1^H NMR Analysis

**Figure 2:**
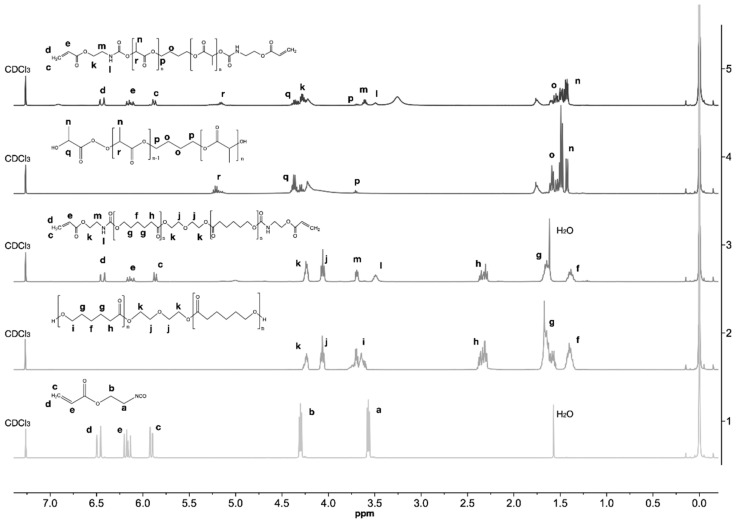
^1^H NMR spectra of (**1**) 2-isocyanoethyl acrylate (AOI) (**2**) polycaprolactone (PCL) (**3**) mPA (**4**) oligomers based on lactic acid (oligLA) (**5**) mLA in deuterated chloroform (CDCl_3)_ at 400 MHz.

^1^H NMR spectra of (1) AOI (2) PCL (3) mPA (4) oligLA (5) mLA are presented in Figure 2. The typical backbone peaks of PCL can be seen (3) at (f) 1.4, (g) 1.7, (h) 2.4 (j) 4.0 and (k) 4.2 ppm, which are assigned to the methylene proton (-C***H***_2_). These peaks are also present in the mPA (4) spectra, except the terminal methylene protons (-C***H***_2_OH) at (i) 3.6 ppm [17,19], due to the reaction of the -OH group with the urethane group from AOI [6] and, therefore, a new peak (l) around 3.5 ppm is present, which is assigned to the urethane groups (-N***H***CH_2_-) [2,6,19]. The mLA (5) spectra follows the same interpretation, presenting the typical oligLA peaks at (r) 5.2 ppm from methine proton -O-C***H***(CH_3_)-C(O)-O-, (n) 1.4 ppm from the methyl group -C*H*_3_ adjacent to the methine proton previously mentioned and (o) 1.5 and (q) 4.3 ppm assigned to the methylene proton (-C***H***_2_) in the oligLA backbone [2,6,19], along with the presence of two new signals at (l) 3.5 and (m) 3.6 ppm which were assigned to the urethane groups (-NH-C**H**_2_- and -*N**H**-C=O* protons), respectively [6,18,19], resulting from the reaction of the oligLA hydroxyl groups with the AOI urethane groups. Moreover, in both mLA (5) and mPA (4) spectra, the three resonance peaks are present at (c) 5.9, (e) 6.2 and (d) 6.5ppm, assigned to the olefinic protons of AOI, confirming the success of the synthesis.

### 3.3. Rheological Studies

**Figure 3:**
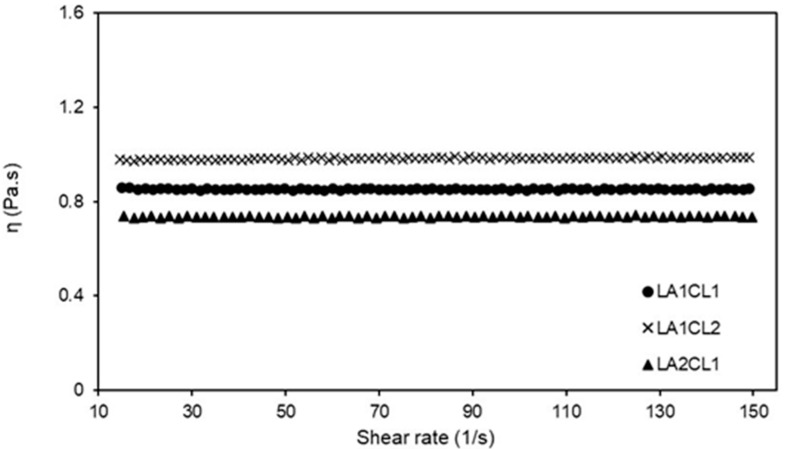
Viscosity of the three blends as function of shear rate, at constant temperature (25 °C).

Rheological studies were carried out in order to establish the relation between viscosity and shear rate (Figure 3). This analysis was performed considering the intended conditions for materials application (at room temperature).

Figure 3 shows the Newtonian behavior of the different blends—the viscosities are constant with the increase in the shear rate, meaning that the viscosity will remain constant no matter how fast they are forced to flow through any channel.

The similarity in flow behavior between the materials indicates that there are slight changes in their molecular structures. Additionally, all blends have relatively low viscosities, with values between 0.7 and 1 Pa.s, with LA2CL1 being the one with the lowest shear viscosity value. The results herein obtained are in agreement with the values previously reported in literature, including the tests performed on commercial bioadhesives [20,21].

Considering the application as surgical adhesives, the prepared blends must be easy to apply and adapt to the injured tissue. Therefore, the low viscosity (and a Newtonian profile) of these materials will allow them to flow and spread when acted upon by gravity or some other type of force, facilitating its application by a variety of methods.

### 3.4. Dynamic Contact Angles

**Figure 4:**
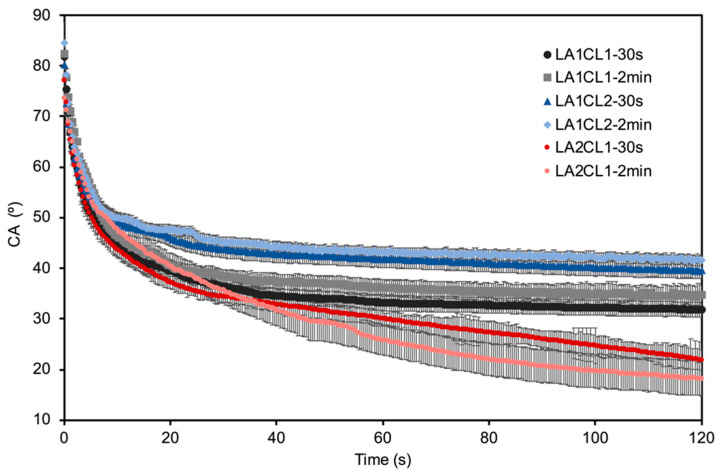
Dynamic water contact angles profiles obtained for the different adhesive formulations.

Figure 4 shows the registered dynamic water contact angle results for the different blends after 30 s or 2 min of crosslinking reaction time. The results revealed that all WCA started between 75° and 85°, showing a slightly hydrophobic behavior of all blends. Furthermore, the crosslinking time did not influence the water affinity of the blends, since no significant differences in the water spreading could be registered between the different crosslinking reaction times. However, some differences can be noticed concerning the composition of the blends. The evidence of mCL and mLA amount in the blends composition can be perceived from the obtained results. The adhesives with higher amount of mCL showed to have a less hydrophilic surface, with a final WCA of around 43°, due to PCL’s strong hydrophobic nature [20]. On the other hand, the adhesives with higher amount of mLA (LA2CL1) presented lower WCA (around 20°) due to the presence of the hydrophilic OH groups from LA [2], with the tendency to decrease, while the adhesives LA1CL1 and LA1CL2 presented a stable WCA of around 40° and 35°, respectively, after 35 s. Therefore, the presence of a higher amount of mLA influences the biological response of these materials.

### 3.5. Gel Content

The analysis of the gel content was performed to assess the crosslinking degree of the films. Results of 100% gel content represent a complete crosslinking in which C=C were fully converted.

The crosslinking time is a determining aspect in the crosslinking degree results. Due to that, in this study, the influence of two different UV exposure times on the different prepared adhesive films was evaluated (Table 1).

As would be expected, Table 1 shows that higher UV exposure periods result in higher percentages of gel content as well as in stiffer and more compact materials.

Additionally, it was possible to verify that increasing mCL content in the polymeric blend (LA1CL2) led to an increase in gel content and, therefore, an enhanced crosslinking degree. This higher crosslinking and stability of the mCL urethane matrix linkages is due to its more pronounced rigidity and to the stronger establishment of hydrogen bonds between urethanes NH and CO/C=O groups in the hard segments of the mCL backbone in the urethane.

### 3.6. Hydrolytic Degradation

**Figure 5:**
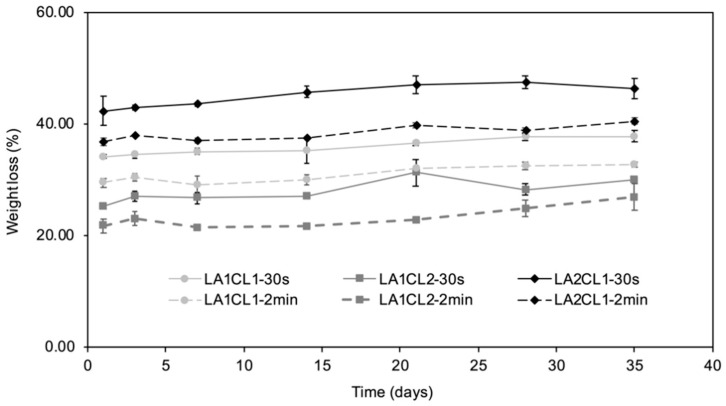
Weight loss (%) of the produced adhesives over 5 weeks.

The biodegradability and capacity of the surgical adhesives being absorbed by the organism is an important property when they are aimed to be used in internal applications [6,21]. To assess the hydrolytic degradation of the photocrosslinked materials, three samples of each adhesive were placed in PBS (pH 7.4) and incubated at 37 °C, to replicate physiological conditions. The assay was carried out for 5 weeks, and the weight loss was calculated over time, which is presented in Figure 5.

All adhesives showed greater weight loss in the first 24 h of testing. This result can be explained by the release of residual solvent (diethyl ether) still present in the matrix, as well as unreacted molecules from the UV-curing reaction [2,6].

The hydrophobicity and stability of PCL makes the mCL macromer more resistant and stable than mLA macromer, whose ester bonds are more susceptible to hydrolytic degradation [21,22]. These results are also related to the WCA (Section 3.4), which showed that adhesives with a higher amount of mLA presented lower WCA and, consequently, a more hydrophilic surface. As can be observed in Figure 5, the increase in the proportion of mLA in the adhesives resulted in higher weight loss percentages. LA2CL1 adhesive presented the higher degradation value: 46.39% ± 1.75% for 30 s irradiation. On the contrary, LA1CL2-2min presented the lowest weight loss degree (26.82% ± 2.40%). All tested adhesives showed higher percentages of degradation for shorter irradiation times, due to lower degree of crosslinking.

Although all the materials have potential to be used as surgical adhesives, the LA1CL1 and LA1CL2 formulations were the ones that presented the most satisfactory mass loss results for both photocrosslinking times (30 s and 2 min).

### 3.7. Thermal Properties

**Figure 6:**
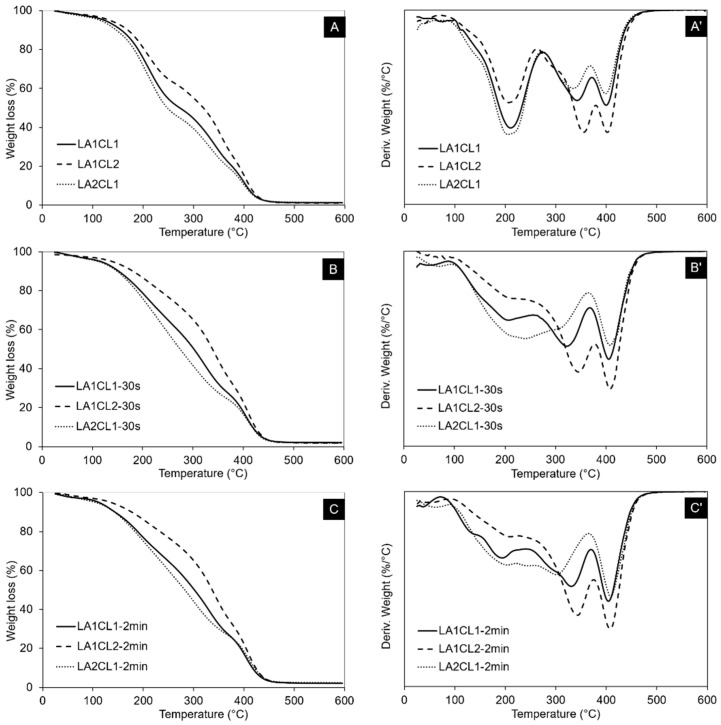
Thermograms registered for the blended macromers (**A**) and their crosslinking products obtained with 30 s (**B**) and 2 min (**C**) of UV irradiation. Figures **A’**, **B’** and **C’** show the correspondent derivative thermogravimetry (DTG) thermograms.

Thermal stabilities of initial blended macromers as well as their crosslinked films obtained with 30 s or 2 min UV irradiation are presented in Figure 6. Furthermore, a summary of the main registered thermal events is presented in Table 2.

As presented in Figure 6A,A’, thermal degradation of LA1CL1 presents an intermediate profile between de LA1CL2 and LA2CL1. Moreover, all blends present a three stage degradation profile. The first one is related with the degradation of the urethane bonds and subsequent formation of carbon dioxide and monoxide, amines and aldehydes [23]. The second step is ascribed to the degradation of ester bonds in the urethanes’ soft segments and occurred at a higher temperature value for the blend with the larger mCL ratio (L1CL2). This increase in thermal stability is justified by the urethane hard segments in the polymeric backbone, which allow the urethanes’ NH and CO/C=O groups to establish hydrogen bonds [24]. The more rigid structure of PCL and stronger hydrogen bonding contribute to the increased thermal stability of the system. Finally, the last degradation step is attributed to the decomposition of C=O, C=C, C–O and C–H bonds which present higher energies and, therefore, require a larger temperature value to degrade [23].

The increased thermal stability with mCL is also visible in the crosslinked blends. For both UV irradiation times, it was verified that thermal degradation occurs at higher values of temperature, when compared with the blends with lower mCL ratio. Moreover, all crosslinked materials present higher thermal stability than the corresponding original blend. As can be perceived in Figure 6B’,C’, the areas under the curves of the first degradation step are significantly lower than the ones of the original blends (Figure 6A’), meaning that the crosslinked structure of the polymeric blends contributes to increasing the stability of the polymeric structural bonds.

Considering the temperature at which samples’ weight decreased into 50% of the initial one (T_50_ wt%), similar findings were observed. Herein, the blends containing a higher mCL ratio are more thermally stable, presenting degradation temperatures between 30 and 60 °C above the other formulations.

Moreover, at 500 °C, all the materials were almost fully degraded with a medium residue value of 1.35% for the initial blends and of 2.23% for the crosslinked ones. Additionally, no significant differences were observed in this parameter for the two UV irradiation periods tested.

### 3.8. Characterization of the Crosslinked Polymeric Blends’ Biological Properties

#### 3.8.1. Cell Growth and Proliferation

**Figure 7:**
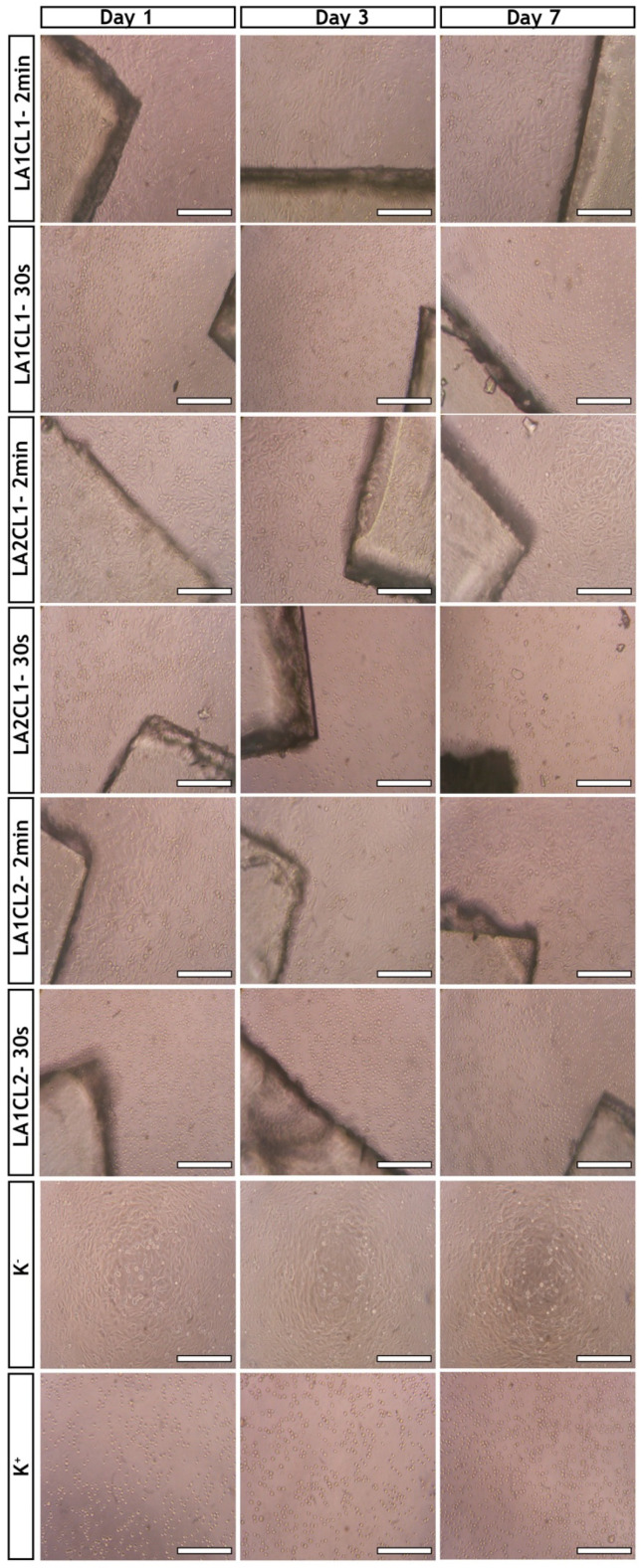
Optical microscopic images of NHDF cells cultured in the presence of produced films (LA1CL1, LA1CL2 and LA2CL1 photocrosslinked for 2 min and 30 s) for 1, 3 and 7 days; K^−^ (negative control); K^+^ (positive control). Scale bar represents 200 µm. Magnification 100×; resolution 1.2 µm.

The NHDF cells were seeded in contact with films and then the optical microscopes images were acquired after 1, 3 and 7 days of incubation, as can be observed in Figure 7.

Morphology of the human fibroblast cells did not show any variation after being incubated with 2 min UV irradiated films (LA1CL1-2min, LA1CL2-2min and LA2CL1-2min) for at least 7 days. However, the cells seeded in contact with films crosslinked during 30 s displayed a morphology similar to the ones of K^+^ (dead cells treated with ethanol) revealing that these specific films (LA1CL1-30s, LA1CL2-30s and LA2CL1-30s) induced a cytotoxic effect on the tested cells.

**Figure 8:**
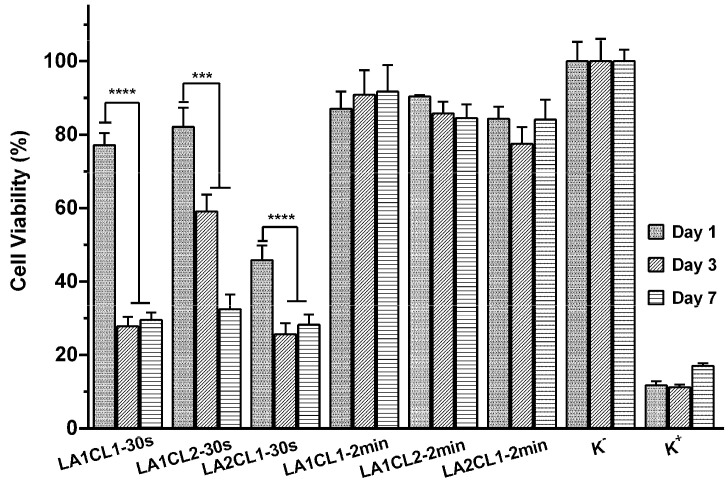
Characterization of cell viability through the MTT assay after 1, 3 and 7 days of incubation. K− (negative control); K+ (positive control). Data are presented as the mean ± standard deviation, *n* = 5, *** *p* < 0.001, **** *p* < 0.0001.

Furthermore, the cell viability was also quantified through the MTT assay at the same timepoints. Through this assay, it was noticed that the absorbance of the samples is proportional to the amount of the purple formazan crystals produced by the viable cells in each well [15,20]. The results obtained (Figure 8) clearly demonstrate that the UV irradiation times influenced the biocompatible character of photocrosslinked films. Therefore, 30 s irradiation time can be insufficient to promote an effective crosslinking between mLA and mCL, thus promoting the release of the unreacted molecules and residual solvent (diethyl ether) which induce a cytotoxic effect for fibroblasts cells. Such findings are in agreement with the results obtained in the determination of gel content and weight loss (Section 3.5 and Section 3.6).

Considering these results, the films photocured for 2 min exhibited an improved biological profile to be used as bioadhesives. The blending between mLA and mCL allowed flexible and stable films with a swelling and hydrophilicity profile to be obtained that promote the cell adhesion and proliferation. These are quite important results, since exposure time is critical when working with UV irradiation. Extended UV irradiation periods of time may result in cell mutation or even death. However, the results herein presented prove that a maximum of 2 min of UV irradiation does not have a negative impact on cell viability. The same fact has been reported by Sabnis et al., who showed that exposure of muscle cells in the presence of Irgacure^®^ 2959 to 1, 3 and 5 min of UV irradiation did not result in any statistically significant decrease in cell survival rates comparing to control cells [25].

#### 3.8.2. Antimicrobial Activity

When a surgical adhesive is aimed to improve the wound closure process, it is extremely important that the material avoid the occurrence of a severe and devasting skin infection [26]. In this work, the antimicrobial profile of the films was assessed against *E. coli* (Gram-negative bacterium) and *S. aureus* (Gram-positive bacterium). The films were placed in contact with bacterial culture, and the diameters of the inhibitory halos were determined after 24 h of incubation. In addition, the bacterial colonization on the surface of the films was also characterized by SEM technique.

**Figure 9:**
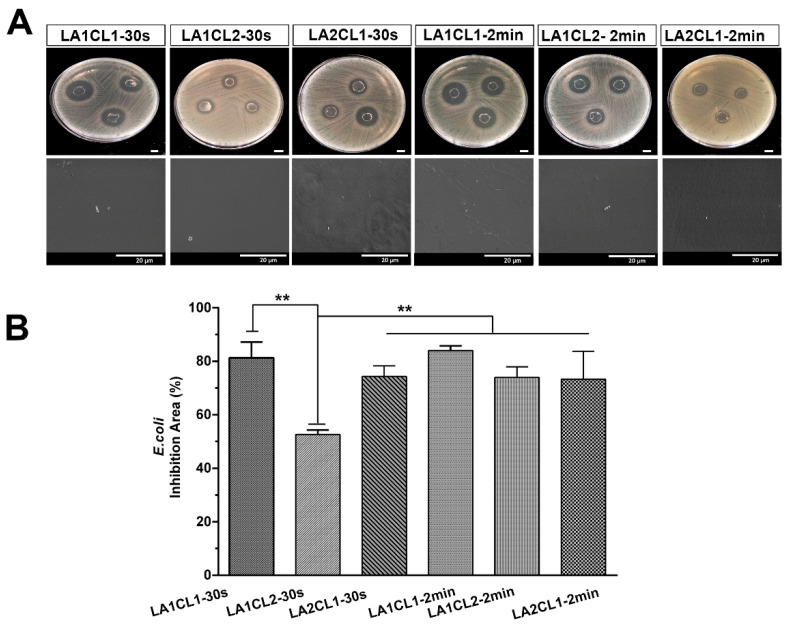
Assessment of antimicrobial profile of the photocrosslinked films against *E. coli*. (**A**) Macroscopic images of the inhibitory halos. (**B**) Relative value of the inhibition area presented by the films. Data are presented as the mean ± standard deviation, *n* = 3.

The results presented in Figure 9 clearly evidenced that all formulations displayed a high antimicrobial effect against *E. coli* growth, exhibiting an inhibition area higher than 70%, with the exception of the LA1CL2-30s formulation that displayed a lower inhibition area with a value of 52.63% ± 1.68%.

**Figure 10:**
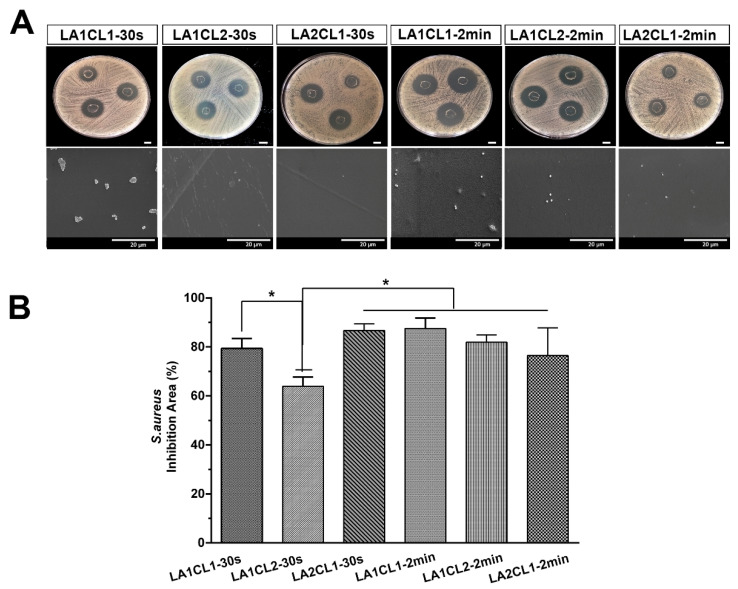
Assessment of antimicrobial profile of the photocrosslinked films against *S. aureus*. (**A**) Macroscopic images of the inhibitory halos. (**B**) Relative value of the inhibition area presented by the films. Data are presented as the mean ± standard deviation, *n* = 3, * *p* < 0.05.

Similar results were also observed when the adhesives were incubated with *S. aureus* (Figure 10), where higher inhibition area values (>75%) were noticed for all adhesives, with lower inhibition area value (63.95% ± 3.77%) exhibited by LA1CL2-30s formulation. Such results were expected since this formulation presents a higher amount of mCL and PCL is known by its hydrophobicity and non-antimicrobial activity [27,28].

According to the SEM images, the bacterial colonization of *E. coli* and *S. aureus* were effectively suppressed at the surface of all prepared films. In general, all formulations presented an excellent antimicrobial effect, which can be correlated with the hydrophilic character of the adhesives due to the presence of mLA. According to the literature, the hydrophilic surfaces (WCA > 40°) impair the bacterial growth, since the adsorption of the water molecules on these surfaces is favorable, promoting repulsive forces of proteins, which reduces the protein adsorption [29]. Indeed, Yuan et al. verified that a superhydrophilic substrate presented a limited bacterial binding, due to the repulsive interaction between *E. coli* and the substrate [30]. Furthermore, it is also described that the lactic acid oligomers are able to disrupt the cytoplasmic membranes, the content and activity of bacterial proteins, leading to bacterial death [31,32].

## 4. Conclusions

In the present work, mCL and mLA blends were prepared with different proportions and UV cured for 30 s and 2 min. Generally, the obtained polymeric blends presented a suitable viscosity to be used as bioadhesives while the crosslinked films showed to be flexible yet resistant. However, different proportions led to distinct behavior of the films. LA2CL1 presented a less crosslinked matrix, a more hydrophilic surface and higher degradation profile. On the other side, the UV irradiation time influenced the biocompatible character of the films. In general, the films photocrosslinked for 2 min (LA1CL1-2min, LA1CL2-2min and LA2CL1-2min) displayed a better biological performance, since the human fibroblasts remain viable and proliferated for 7 days, when seeded in contact with these materials. Moreover, the antimicrobial assays revealed that all photocured films exhibited an excellent antimicrobial effect against *E.coli* and *S. aureus* growth, with the exception of LA1CL2-30s formulation that presented the lower inhibition area values (<70%).

Considering all obtained data, the films, especially those crosslinked under UV irradiation for 2 min, presented the most auspicious properties to be applied as bioadhesives.

## Figures and Tables

**Table 1:** UV exposure times (s) and corresponding gel content for each crosslinked adhesive.

Adhesive Blends	UV Irradiation Time (s)	Gel Content (%)
LA1CL1	30	64.2 ± 3.40
	120	77.6 ± 0.27
LA1CL2	30	68.9 ± 0.45
	120	79.5 ± 0.31
LA2CL1	30	47.8 ± 1.78
	120	68.1 ± 0.14

**Table 2:** Summary of the main registered thermal events in thermo gravimetric analysis (TGA). Tmax represents the temperature value corresponding to the highest degradation rate at each degradation stage and ΔW represents the correspondent weight loss (%). T50 represents the temperature at which samples’ weight decreased to 50% of the initial one.

	1st Stage		2nd Stage		3rd Stage			
	Tmax1 (°C)	ΔW1 (wt %)	Tmax2 (°C)	ΔW2 (wt %)	Tmax3 (°C)	ΔW3 (wt %)	T50 wt % (°C)	Residue at 500 °C (wt %)
LA1CL1	209.32	28.87	347.00	70.82	404.91	90.08	272.69	1.34
LA1CL2	207.41	21.92	357.99	66.00	405.57	87.40	319.50	1.17
LA2CL1	206.91	31.83	333.83	70.65	400.09	89.53	247.79	1.55
LA1CL1-30s	206.30	23.00	320.66	57.02	406.49	85.00	301.83	2.21
LA1CL2-30s	216.83	16.52	348.44	54.78	410.88	83.30	338.78	2.02
LA2CL1-30s	213.18	20.51	312.46	62.36	405.70	85.58	274.41	1.99
LA1CL1-2min	194.07	21.45	330.04	60.39	404.24	84.65	301.66	2.29
LA1CL2-2min	209.76	15.67	342.45	52.75	407.79	81.94	337.04	2.23
LA2CL1-2min	203.30	25.90	300.66	56.05	408.31	84.93	282.24	2.56
